# BRAF K601E Mutation in Oncocytic Carcinoma of the Thyroid: A Case Report and Literature Review

**DOI:** 10.3390/jcm12226970

**Published:** 2023-11-07

**Authors:** Antonio Matrone, Fabrizia Citro, Carla Gambale, Alessandro Prete, Elisa Minaldi, Raffaele Ciampi, Teresa Ramone, Gabriele Materazzi, Liborio Torregrossa, Rossella Elisei

**Affiliations:** 1 Unit of Endocrinology, Department of Clinical and Experimental Medicine, Pisa University Hospital, 56124 Pisa, Italy; fabriziacitro93@gmail.com (F.C.); carla.gambale@phd.unipi.it (C.G.); alessandro.prete22@gmail.com (A.P.); elisaminaldi@hotmail.it (E.M.); raffaele.ciampi@unipi.it (R.C.); teresa.ramone@med.unipi.it (T.R.); rossella.elisei@med.unipi.it (R.E.); 2 Endocrine Surgery Unit, Department of Surgical, Medical, Molecular Pathology and Critical Area, Pisa University Hospital, 56126 Pisa, Italy; gabriele.materazzi@med.unipi.it; 3 Department of Surgical, Medical, Molecular Pathology and Critical Area, Anatomic Pathology Section, Pisa University Hospital, 56126 Pisa, Italy; libo.torregrossa@gmail.com

**Keywords:** oncocytic carcinoma of the thyroid, Hürthle cell, BRAF K601E, next generation sequencing, metastatic thyroid cancer, actionable mutation

## Abstract

Background: Thyroid carcinoma (TC) is the most common endocrine cancer, with papillary thyroid carcinoma (PTC) being the most common subtype. BRAF and RAS oncogene were characterized as the most frequently altered genes in PTC, with a strong association between genotype and histotype. The most common mutation in BRAF gene is V600E and is prevalent in classic and aggressive variants of PTC, while BRAF K601E mutation is the most common among the other rare BRAF mutations. BRAF K601E mutated thyroid carcinomas are usually characterized by low aggressiveness, except for anecdotal cases of poorly differentiated TC. Case presentation: We described a case of oncocytic carcinoma of the thyroid (OCA) with an aggressive clinical course, including widespread metastasis and resistance to radioiodine treatment. Molecular analysis revealed the exclusive presence of the BRAF K601E mutation in both primary tumor and metastatic lesions. Accordingly, a revision of the literature about aggressive TC cases carrying BRAF K601E mutation was performed. Conclusion: Although rare, this case emphasizes the relevance of considering BRAF K601E mutation in advanced non-PTC thyroid carcinomas, since it can be considered an actionable mutation for target therapies.

## 1. Introduction

Thyroid carcinoma (TC) is the most common endocrine neoplasia, accounting for about 3.8% of all human cancers. Papillary thyroid carcinoma (PTC), a well-differentiated thyroid carcinoma (DTC) with papillary histologic architecture, represents about 80% of all TC [1]. PTC usually shows a less aggressive clinical behavior, with a higher rate of cure over time, although several cases can develop lymph nodes and distant metastases both at the time of diagnosis and during the follow-up [2].

Beyond histologic architecture, immunohistochemistry is an essential complement to standard histological methods able to provide additional information about cells’ origins and allow a more detailed cellular characterization [3]. It is primarily recommended to better identify lesions that do not appear to originate from follicular thyroid tissue (i.e., medullary thyroid carcinomas, lymphomas, parathyroid lesions or neoplasms, sarcomas, metastases from other organs, etc.) or do not have (i.e., carcinoma showing thymus-like differentiation—CASTLE) [4] or have lost (i.e., anaplastic thyroid carcinoma) [5] the classic architecture of thyroid tissue.

For several years, PTC has also been widely studied to characterize its molecular profile. Recently, a comprehensive approach with a next generation sequencing (NGS) platform identified the two most frequent altered oncogenes in PTC: BRAF and RAS, whose point mutations (i.e., only BRAFV600E for BRAF, several for the 3 RAS genes) are mutually exclusive [6]. Both BRAF and RAS oncogenes are involved in the mitogen-activated protein kinase pathway signaling (MAPK), necessary for the regulation of cell functions including proliferation, gene expression, differentiation, mitosis, cell survival, and apoptosis [7]. Interestingly, a strong correlation between genotype and histotype has been described. Indeed, while BRAF V600E mutation is highly prevalent in a classic and aggressive variant of PTC like tall cell, RAS mutations are more common in the follicular variant of PTC (FV-PTC). Likewise, the genotype is correlated to cancer behavior since tumors carrying BRAF V600E mutation are characterized by a low expression of the thyroid differentiation genes and a higher prevalence of cases refractory to radioiodine (^131^I) treatment, when compared with those carrying RAS mutation [6].

It is worth noting that the mutational landscape of DTC, particularly PTC, can also be different according to the different populations studied. Indeed, mutations in PNPLA5, a gene closely associated with lipid metabolism, have been described as driver mutations in PTC detected in the Chinese population such as BRAF mutations, different from that observed in Western countries [8].

Whether V600E is the most common mutation involving the BRAF gene, other rarer BRAF mutations (r-BRAF) have been described showing a RAS-like clinical behavior [9]. Among r-BRAF mutations, the most common (64%) involves the amino acid substitution of lysine with glutamic acid at codon 601 (p.K601E). This mutation is associated with lesser oncogenic activity than V600E. In vitro studies showed that BRAF kinase activity resulting from K601E mutation is 2.5 times lower if compared with that derived from the V600E mutation [10]. This is so true that another author postulated that in the scenario of thyroid nodules having cytology suspicious for malignancy, the mutational pattern detected in fine needle aspirates can influence the surgical procedures, from hemithyroidectomy up to total thyroidectomy and lateral-cervical lymph nodes dissection [11]. Indeed, the absence of BRAF V600E and TERT mutations and the presence of at least one of the mutations associated with less aggressive behavior such as BRAF K601E, other RAS-like mutations and PAX8/PPARγ rearrangement carried the lower risk of recurrence (<10%), and then could be safely managed with hemithyroidectomy. Conversely, in the presence of the BRAF V600E mutation, particularly if in combination with other mutations such as TERT, a more extensive surgery is suggested due to the higher risk of recurrence over time (from 10 to >40%) [11].

This is consistent with the evidence that the BRAF K601E mutation has even been described in cases of follicular adenomas, microPTC and noninvasive follicular thyroid neoplasm with papillary-like nuclear features (NIFTP), but mainly occurs in FV-PTC with a quite indolent course [9,12,13,14]. Of note, the prevalence of BRAF K601E mutation in FV-PTC is approximatively 6%, resulting as the third most frequent mutation detected in this variant of PTC, after BRAF V600E and NRAS [15].

In multifocal PTC, different BRAF mutations (V600E and K601E) in different thyroid cancer foci characterized by different histological variants, within the same thyroid gland, have been described: in most cases, K601E mutation was found in foci with less aggressive clinical behavior such as FV-PTC, while in the same gland V600E was mainly associated with the classic variant [16], tall cell variant or poorly differentiated thyroid carcinoma (PDTC) [17]. However, within the same gland, in one case of tall cell variant PTC carrying BRAF V600E mutation, the expression of BRAF K601E mutation was also found in a focus of PDTC [14].

In Hürthle cell carcinoma, BRAF K601E mutations have never been described so far. Since recently, in the fifth edition of the World Health Organization’s classification of tumors of endocrine organs, it is recommended to replace the wording Hürthle cell thyroid carcinoma with oncocytic carcinoma of the thyroid (OCA) [18], so throughout the text when we referred to Hürthle cell thyroid carcinoma, we used the term OCA.

Herein we described a clinical case of OCA characterized by very aggressive clinical behavior, carrying the BRAF K601E mutation as the only driver mutation detected. To our knowledge, this is the first described case of OCA of thyroid gland carrying BRAF K601E mutation. We also provided a review of the current literature available about BRAFK601E mutations in follicular thyroid carcinoma (FTC) and PDTC.

## 2. Materials and Methods

### 2.1. Study Design and Search Strategy

We conducted a systematic literature search in the Scopus, MEDLINE, and ScienceDirect databases to identify all relevant studies published up to October 2023. We used the following search terms: (“BRAF K601E” OR “BRAFK601E”) AND (“Hürthle cell thyroid carcinoma” OR “Oncocytic thyroid carcinoma” OR “Follicular thyroid carcinoma” OR “poorly differentiated thyroid carcinoma” OR “Hürthle cell thyroid cancer” OR “Oncocytic thyroid cancer” OR “Follicular thyroid cancer” OR “poorly differentiated thyroid cancer”). We manually searched the references cited in all full-text papers to identify additional studies for inclusion. The literature search was performed according to the Preferred Reporting Items for Systematic Reviews and Meta-Analysis (PRISMA) guidelines.

### 2.2. Inclusion and Exclusion Criteria

We included any study reporting cases of BRAFK601E mutations in OTC, FTC, and PDTC, regardless of study design. Studies reporting BRAFK601E mutations in PTC were not considered. We excluded records in languages other than English, although studies in other languages could be considered if they included an English abstract. Two independent reviewers (EM and FC) evaluated the eligibility of each selected paper, with a third reviewer (AM) available to resolve any disagreements.

### 2.3. Data Extraction

The following data were extracted from the selected papers: study information (authors, year of publication), patient’s age and sex, primary tumor histology, other relevant histologic features and dimension of the tumor, presence of lymph nodes and distant metastasis, and other mutations harbored by the tumor. Data were retrieved independently by two authors (EM and FC) and confirmed by a third reviewer (AM).

## 3. Results

### 3.1. Case Report

#### 3.1.1. Clinical Presentation and Initial Treatments

In 2003, a 63-year-old Caucasian man showed a right neck nodule of about 3 cm, diagnosed as benign nodule after cytological examination on fine needle aspiration (FNAC). The patient performed no more clinical and ultrasonographic evaluations over time. However, in 2010, due to the progressive increase in size of the right nodule (8 cm of maximum diameter) and evidence of another nodule in the left lobe (6 cm of maximum diameter) associated with onset of cervical pain, FNAC was repeated and showed a suspicious follicular lesion with oncocytic cells in both nodules. Therefore, a total thyroidectomy with prophylactic central compartment lymph nodes dissection was performed. Histology revealed a widely invasive bilateral oncocytic thyroid carcinoma, with infiltration of soft perithyroidal tissues and muscles, neoplastic emboli (number > 4), without lymph node metastasis (pT3mN0Mx according to TNM 7th edition). After surgery, a total activity of 331 mCi (12.2 GBq) of ^131^I was administered in three times for the presence of a right latero-cervical lymph node metastases (max diam 15 mm) detected by ultrasound and cytologically confirmed at FNAC, and small bilateral lung metastasis (maximum diameter 5 mm, characterized by CT scan), who showed radioiodine uptake. However, after the third ^131^I treatment, no radioiodine uptake was described anymore at the post-therapy Whole Body Scan (WBS) despite the persistence of the metastatic lesions.

Despite the recommended schedule of clinical, biochemical, and imaging evaluations every 6–12 months, the patient did not adhere to these medical guidelines. Instead, he returned to our institution after a gap of six years, complaining of a progressive increase in a right lateral-cervical mass and frequent episodes of hemoptysis.

#### 3.1.2. Progression of Disease and Subsequent Treatments

Physical examination revealed a non-tender, fixed, nodular mass of about 3.5 cm in the right latero-cervical region. Serum thyroglobulin levels were significantly increased compared to those evaluated 6 years before (15,646 vs. 26.35 ng/mL).

**Figure 1:**
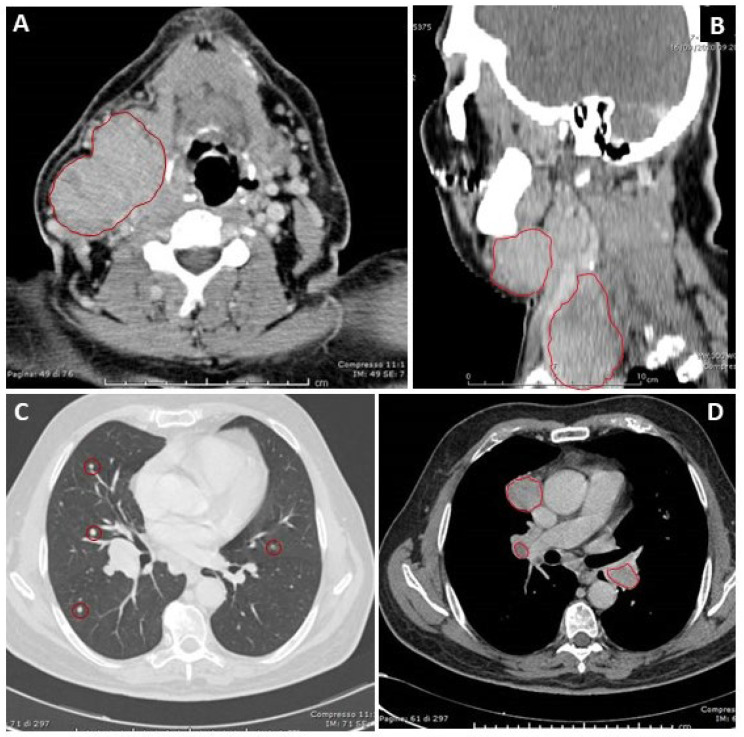
Representative images of contrast-enhanced total body CT: (**A**) Multiple confluent nodular lesions in right latero-cervical region, of overall size 56 × 58 mm, reaching superficial structures of neck and invading peri-thyroid muscles. (**B**) The nodular lesion in supraclavicular region (47 × 35 mm) invading muscles, causing compression and likely invasion of the internal jugular vein, and displacing anteromedially the right common carotid artery. (**C**) Multiple metastatic nodular lesions at midfields of lungs bilaterally. (**D**) Ilo-mediastinal lymphadenopathies (largest of 33 × 37 mm).

Neck US showed three suspicious metastatic lymph nodes (53 mm, 13 mm and 20 mm) in the right latero-cervical region. A contrast-enhanced total body CT scan confirmed the presence of multiple confluent suspicious lymph nodes metastases, the largest of 58 mm, with the involvement of adjacent muscles and reaching neck superficial structures and another lesion of 47 mm in the right supraclavicular region, which determined compression and invasion of the internal jugular vein. Moreover, multiple nodular lesions in lungs and some mediastinal suspicious lymphadenopathies (the largest of 37 mm) were described in the thorax (Figure 1).

**Figure 2:**
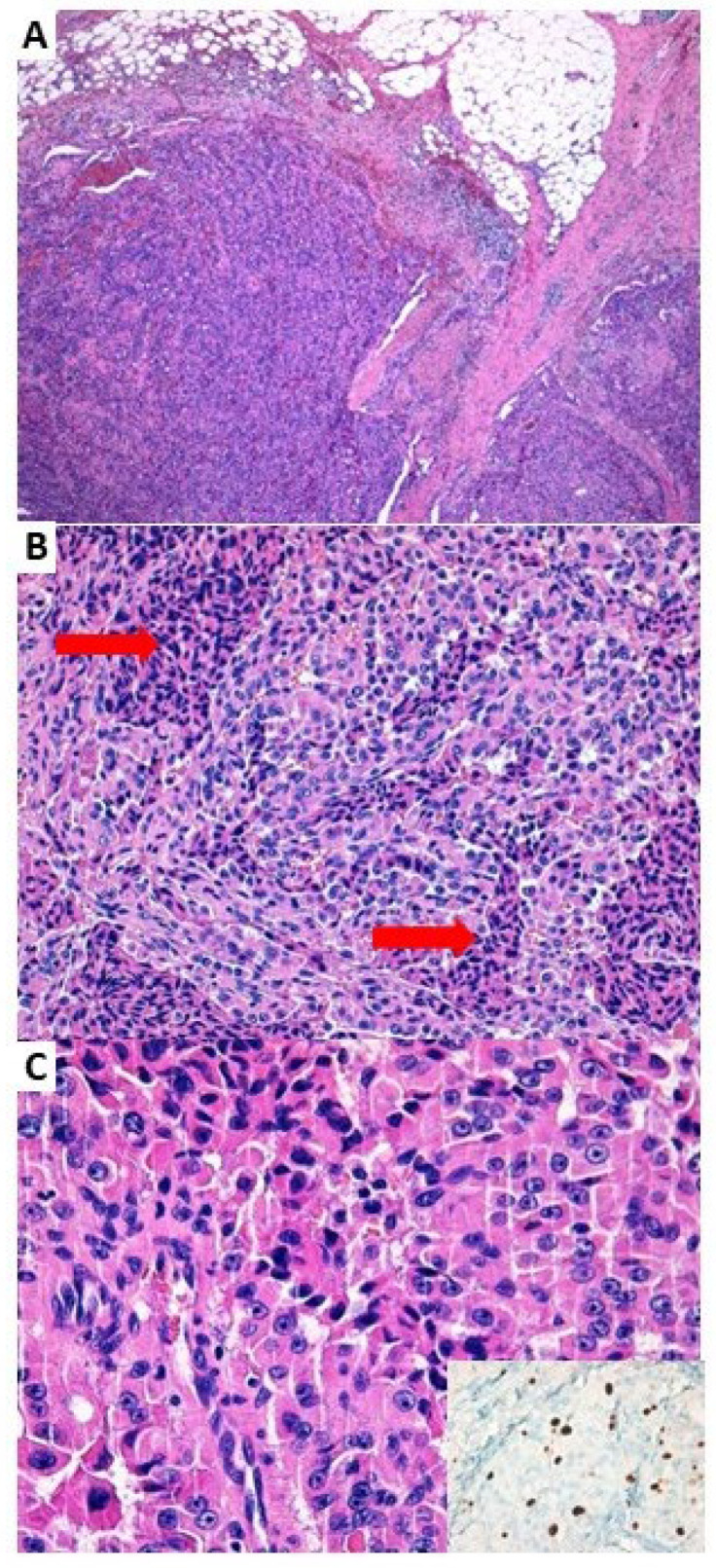
Representative histopathological images of a lymph node metastasis. (**A**) The metastatic lesion totally replaces the lymph node and shows extra-nodal extension (H&E, original magnification ×40). (**B**) The neoplasm shows histologic features of poorly differentiated thyroid carcinoma, with prevalent solid and trabecular growth and incipient necrosis, defined as clusters of cells showing nuclear pyknosis, karryhorexis, and cytoplasmic (see arrows, H&E, original magnification ×200). (**C**) At major magnification, the oncocytic cell morphology is still recognizable, with abundant oxyphil cytoplasm and prominent nucleoli (H&E, original magnification ×400). In the insert, the proliferative activity, evaluated by immunohistochemistry for Ki-67, is about 10%.

Due to the absence of radioiodine uptake at the previous treatment, the patient was considered refractory to radioiodine and according to the clinical evolution, after excluding potential contraindications, surgical treatment in the right lateral-cervical region was performed. Histology highlighted the presence of metastases of poorly differentiated thyroid carcinoma (PDTC) (defined according to the Turin proposed criteria [15]) but with oxyphilic features, in 4 on 24 removed lymph nodes (max diam 75 mm), with extra nodal extension (ENE) (Figure 2).

Total body CT scan performed 6 months after surgery showed the persistence of the disease in the right lateral-cervical (16 mm) and paratracheal region (32 mm), and the slight increase in size of the mediastinal and lung metastasis. Due to the presence of multiple comorbidities (chronic liver disease with thrombocytopenia, uncontrolled arterial hypertension, and risk of trachea-esophageal fistula), systemic therapy with multikinase inhibitors was not performed and in February 2022 the patient was treated with external radiotherapy (30 Gy) in the neck region. The opportunity to perform external radiotherapy of thoracic lesions was excluded.

At the CT scan, 3 months after neck radiotherapy, a reduction in size of the right lateral-cervical (8 mm vs. previous 16 mm) lesion was documented. At the last follow-up in May 2023, the disease showed no further relevant progression.

#### 3.1.3. Mutational Landscape

Since the aggressiveness of the disease and the peculiar clinical behavior, to evaluate the molecular pattern of this tumor and to potentially find some actionable mutation, a NGS analysis was performed on the metastatic lymph nodes, derived from the second surgery. NGS analysis was performed using a thyroid-specific custom panel for single nucleotide variations (SNV) and small indels included BRAF, RET, H-, K-, N-RAS genes and hot-spot exons of other 12 genes involved in thyroid carcinogenesis, as previously described [19]. Massively parallel sequencing was performed on a 520 Chip for tissue DNA with a GeneStudio S5 deep sequencer (Applied Biosystem—Waltham, MA, USA) and following the manufacturer’s instructions. Analysis was performed using Torrent Suite 5.6 and Ion Reporter 5.14 (Thermo Fisher—Waltham, MA, USA) software with proper bioinformatics workflows as previously described [19]. The nucleotide substitution c.1801A> G (p.K601E) of the BRAF gene was found (allelic frequency: 15.2%). Despite the finding of SNV, that usually is mutually exclusive with other driver mutations, we searched also for RET, NTRK and PAX8/PPAR gamma rearrangements, but without finding any mutation. TERT promoter mutations C288T and C250T were evaluated by direct sequencing as previously described [20], but with a negative result. Moreover, PTEN and TP53 were also evaluated, but again with negative results.

**Figure 3:**
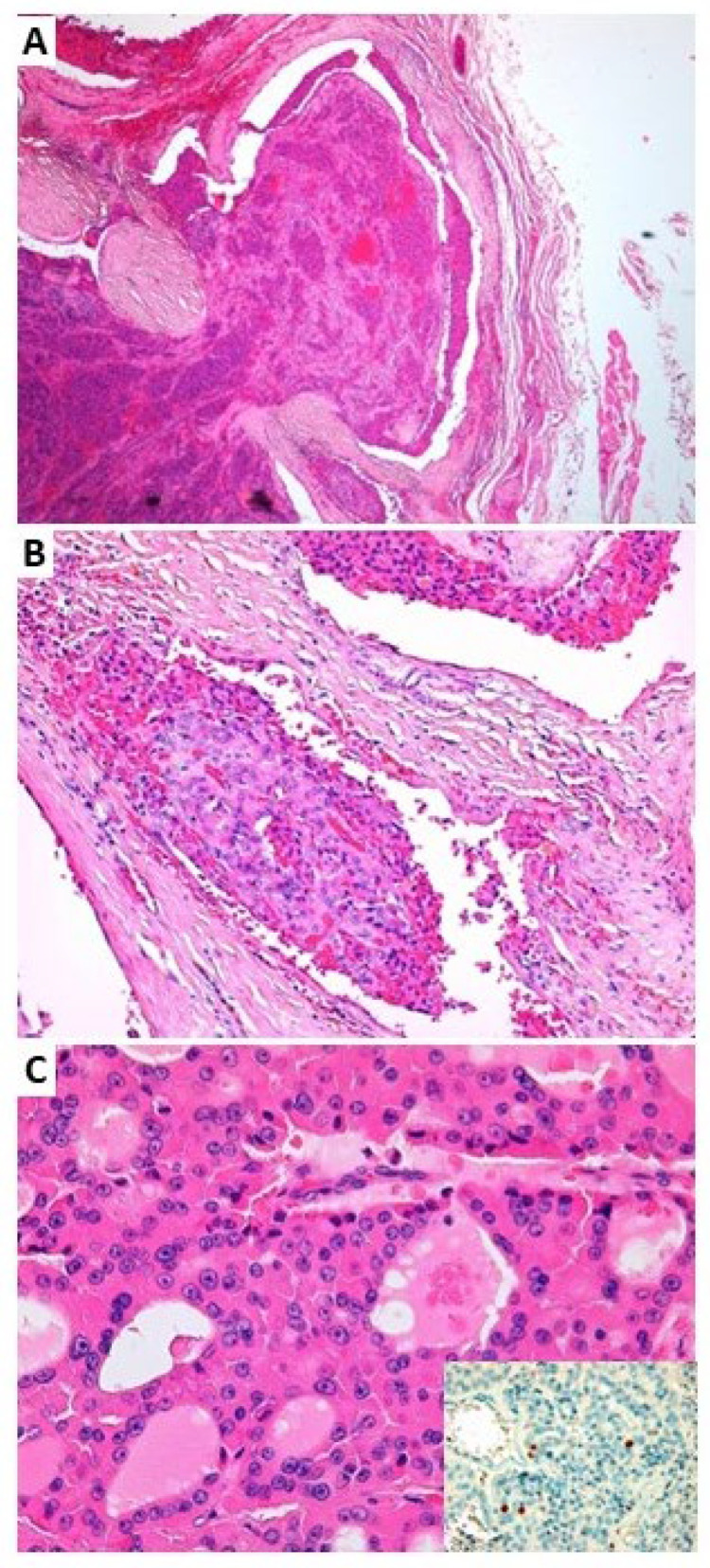
Representative histopathological images of widely invasive oncocytic thyroid carcinoma. (**A**) In this field, the neoplasm invades the entire thickness of the tumor capsule (hematoxylin and eosin (H&E) staining, original magnification ×40). (**B**) A focus of vascular invasion is shown (H&E, original magnification ×200). (**C**) At major magnification, the neoplasm is composed of oncocytic cells with follicular architecture; the neoplastic cells have oxyphil cytoplasm and large centrally located nuclei with prominent nucleoli (H&E, original magnification ×400). In the insert, the proliferative activity, evaluated by immunohistochemistry for Ki-67, is about 3%.

In view of the absence of this mutation in OCA [21], the histology of the primary tumor in both thyroid lobes was re-evaluated. OCA with a mainly solid/trabecular pattern, extensively infiltrating perithyroidal tissues and muscles, with extensive vascular invasion (>4 neoplastic emboli) was confirmed in both primary tumors (Figure 3).

Also, NGS molecular analysis was performed on the primary tumor and the same BRAF K601E mutation was found (allelic frequency: 29.8%), without other molecular alterations in the evaluated genes, including TP53, PTEN and TERT promoter mutations.

### 3.2. Review of the Literature

**Figure 4:**
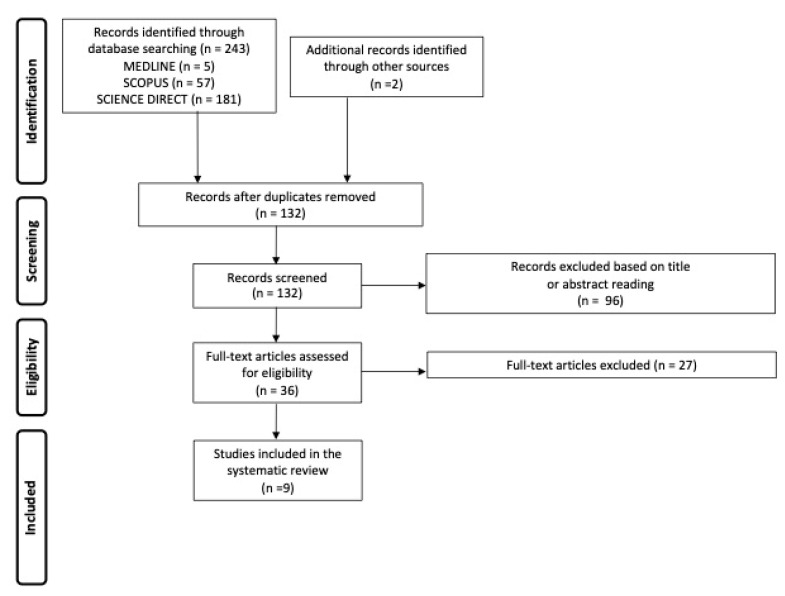
Flow chart showing the study selection process.

We retrieved 243 articles from the search strategy. Two additional records were identified through a manual search of the references cited. After the removal of duplicates (n = 113), 96 records were excluded based on titles and abstracts. The remaining thirty-six full-text articles were assessed for eligibility and at last nine articles met the criteria for data extraction. The flowchart of the study selection is shown in Figure 4.

The data extracted from the included studies are shown in Table 1.

In 2011, Pennelli et al. reported the first case of FTC carrying BRAF K601E mutation [23]. Since then, also when considering large series, few other cases of FTC [12,22,24,25,26], and one PDTC [17] carrying BRAF K601E mutation have been described in detail. Pozdeyev et al. sequenced 779 advanced thyroid carcinomas, including 65 FTC and 35 OTC. They identified three FTC cases with BRAF K601E mutation but none in the OCA [22]. A case of FTC with extensive vascular invasion harboring BRAF K601E mutation associated with TERT C250T mutation was also reported in a series of 199 consecutive thyroid nodules with indeterminate cytology, all surgically treated [12]. Another case of PDTC with the BRAF K601E mutation was found among 69 cases of multifocal PTC [14].

## 4. Discussion

Oncocytic carcinoma of the thyroid (OCA) is defined by WHO as a distinct follicular neoplasm with more than 75% tumor cells with oncocytic features (i.e., hyperchromatic nuclei, prominent nucleoli, and lots of mitochondria making cytopslasm granular and intensely eosinophilic) [18,27], with evidence of malignancy (capsular and/or vascular invasion), without the characteristic nuclear features of PTC and in absence of high-grade features (tumor necrosis and ≥ 5 mitoses per 2 mm^2^) [18,28]. Oncocytic cells are also present in FTC and in the FV-PTC, in the oncocytic subtype of PTC, PDTC and oncocytic medullary thyroid carcinoma, but also in chronic autoimmune thyroiditis [29].

Although these histological features are partially shared from different histotypes of thyroid carcinoma, recent data resulting from molecular studies would support the hypothesis that OCA represents a peculiar histotype of DTC [21]. Indeed, the presence of RAS mutations is rarer if compared with FTC (10%) [22,30] and while in PTC BRAF V600E is the most common mutation (64%), in OCA only four cases carrying BRAF mutation have been described so far. One of the four cases is a mixed tumor, OCA and multifocal PTC with BRAF V600E mutation [31]. The other three cases were identified among a cohort of 41 OCA. They carried missense mutations in the BRAF gene: in two cases p.I300V and in one p.D677N [30]. Conversely, none of the 35 advanced OCA examined by Pozdeyev et al. carried any mutation in the BRAF gene [22].

Common molecular alterations in OCA involve PI3K/Akt/mTOR, a signaling pathway downstream of RAS. Up to 22% of more aggressive OCA, as far as advanced PTC and FTC, carried TERT promoter mutation [32]. TERT is a gene coding for a protein involved in telomere elongation, therefore implicated in cell survival [33]. TERT promoter mutation, although not being considered a driver gene, shows a negative prognostic value since, especially if associated with BRAF mutation, it increases the risk of recurrence up to 8.5 times [34].

Here we described a case of OCA with the BRAF K601E mutation with a peculiar presentation and clinical course. Although the BRAF K601E mutation is mainly detected in low-risk PTC, prevalently in its follicular variant [9,35] with indolent course, our case is characterized by the initial involvement of both thyroid lobes, unlike the typical unifocal presentation of FTC and OCA. Moreover, the disease showed a very aggressive clinical behavior over time, evolving through a dedifferentiation of the tumor, up to the persistence of the disease in the neck and the presence of distant metastatic disease, with loss of the ability to take up the radioiodine.

OCA, such as FTC, may be distinguished in minimally or widely invasive, and some molecular alterations, such as PTEN, TP53 and particularly TERT, are more frequently found in more invasive cancers [36].

In our patient, molecular analysis excluded TERT, TP53 and PTEN mutations in the primary tumor and in the lymph node metastases both carrying only the BRAF K601E mutation.

Therefore, according to the histology and knowledge about OCA, BRAF K601E can be reasonably considered the driver mutation, whereas any other additive molecular alteration could have determined further dedifferentiation and progression towards the PDTC lymph node metastases.

Few reports of thyroid carcinoma other than PTC carrying the BRAF K601E mutation were described in detail (Table 1). Conversely, several other cases, with less on no information about the clinical features, were discovered during genetic analysis of a large series of patients evaluated by PCR-single strand conformational polymorphism followed by DNA sequencing [14], Sanger sequencing [26], direct sequencing [12] or NGS analysis [22] (Table 2).

Some authors have described cases of minimally invasive FTCs, without vascular invasion; conversely, the presence of vascular invasion was reported by others [23,24,25,26,37]. In the FTC case described by Pennelli et al. [23], histology showed the presence of vascular invasion (>4 neoplastic emboli), focal invasion of tumor capsule, solid/trabecular areas, and foci of necrosis, with both well (including some with oxyphilic features) and poorly differentiated cells. Molecular analysis confirmed the presence of BRAF K601E mutation in all areas of neoplasia (follicular, oxyphilic, insular and trabecular areas). However, an activating mutation in PIK3CA was also found. Another case of widely invasive FTC in which BRAF K601E mutation was associated with TERT C250T mutation was described by Censi et al. [12], who analyzed a series of 199 cases of thyroid nodules with indeterminate cytology, all submitted to surgery. Despite the description of the type of cancer, no other information about the clinical course of this case is available. However, in this case, the simultaneous presence of the two genetic alterations can explain the more aggressive behavior of the tumor. At variance, in our case report we did not find any other genetic alteration, at least in the custom panel used in our laboratory.

Three cases of advanced FTC with the BRAF K601E mutation were reported in the Pozdeyev’s data about the molecular alterations in 779 advanced thyroid cancers [22].

Finally, a case of locally invasive and metastatic PDTC with the K601E BRAF mutation was described, in which, after 2 months of therapy with dabrafenib plus trametinib, clinical (thyrotoxicosis), biochemical (increase thyroglobulin levels), metabolic (decreased 18FDG and increased iodine uptake) and histological (decreased Ki-67 and increased expression of NIS, thyroglobulin and TPO) re-differentiation, was observed [14]. Although very rare, the presence of the BRAF K601E mutation in a focus of PDTC was already described in the context of multifocal thyroid cancer within the same gland [14].

Several recent studies highlighted the unique features of the OCA somatic genome compared to other thyroid cancers. Particularly, they confirmed that the mutations found in other thyroid cancers (including RAS, BRAF, RET-PTCs, PAX8- PPARg, etc.) occur only in a small fraction of OCA [27].

Indeed, in a subset of 56 OCA, BRAF mutations was not found, and the frequency of N-RAS was lower than that of FTC [21]. Also, other key mutations detected in PDTC and ATC, particularly the TERT promoter mutation, were found at a lower frequency (22% in OCA vs. 73% in ATC). Conversely, widespread chromosomal losses and a high number of disruptive mutations to protein-coding and tRNA-encoding regions of the mitochondrial genome were recently reported and described as potential genetic drivers in OCA [21,27,30].

It is noteworthy that Ganly et al. [21] described a peculiar set of chromosomic alterations in OCA. They found that a large fraction of OCA showed, on one hand, extensive polysomy, characterized by duplication of whole chromosomes 5 and 7, which causes an increase in the expression of several genes that may provide clonal advantages, particularly in the RAS/RAF/MAPK pathway and in the PI3K/AKT/mTOR pathway. On the other hand, they showed extensive uniparental disomy of the remaining chromosomes, which results in a loss of heterozygosity, potentially promoting the inactivation of many tumor suppressor genes [21].

Since this relatively recent improving in the knowledge of the mutational landscape of OCA, a limitation of the molecular analysis we performed is that we did not study the chromosomal alterations and we cannot fully exclude that other genetic mechanisms able to induce tumor transformation could be involved in this case.

However, this case further shed light on the peculiarity of the mutational landscape of OCA. The presence of a very rare mutation, such as BRAF K601E, usually associated with lesser aggressive thyroid tumors, highlights the importance, at least in peculiar cases like this, to check also for rarer mutations, particularly if specific highly selective drugs have been designed against these mutations.

## 5. Conclusions

BRAF K601E mutation is a rare type of mutation in the BRAF gene, and is more frequently detected in PTC, particularly its follicular variant, in which it is associated with less aggressive presentation and clinical behavior. However, although rarely reported, in other thyroid cancer histology such as FTC or PDTC, it can be associated with various degree of aggressiveness. In our case, for the first time, the presence of BRAF K601E, without TERT, PTEN and TP53 mutation, has been described in an aggressive metastatic OCA. This type of thyroid cancer has been recently better characterized from a molecular point of view, highlighting the low prevalence of the common driver mutations detected in PTC such as BRAF or RAS. However, in advanced cancers different from PTC, including OTC, the presence of BRAF K601E mutation should not be overlooked because it could be detected, albeit with low prevalence, and represents an actionable mutation for which specific drugs are now available.

## Figures and Tables

**Table 1:** Summary of the Follicular Thyroid Carcinomas (FTC) and Poorly Differentiated Thyroid Carcinomas (PDTC) cases carrying K601E BRAF mutation described in detail in the literature.

Histology	Age (Years)	Sex	Relevant Histologic Features	Tumor Dimension (cm)	Lymph Node Metastasis	Distant Metastasis	Treatment	Other Mutations Detected
FTC [22]	78	M	Angio-invasive FTC with poorly differentiated component	8	Absent	N/A	Total thyroidectomy with central compartment lymph node dissection.	Activating mutation in PIK3CA
PDTC [17]	59	F	Poorly differentiated thyroid cancer, with high mitotic count (Ki67:10%). Locally invasive with tracheal invasion	N/A	Absent	Lung, mediastinal and bone metastases	Dabrafenib and trametinib	Absent

N/A: data not available.

**Table 2:** Summary of the Follicular Thyroid Carcinomas (FTC) carrying K601E BRAF mutation, discovered during genetic analysis of large series patients. Little or no information about clinical features is available.

Histology	Age (Years)	Sex	Relevant Histologic Features	Tumor Dimension (cm)	Lymph Node Metastasis	Distant Metastasis	Treatment	Other Mutations Detected
FTC [22]	N/A	N/A	N/A	N/A	N/A	N/A	N/A	N/A
FTC [22]	N/A	N/A	N/A	N/A	N/A	N/A	N/A	N/A
FTC [22]	N/A	N/A	N/A	N/A	N/A	N/A	N/A	N/A
FTC [24]	N/A	N/A	Thick capsule with one focus of capsular and vascular invasion	2	Absent	N/A	N/A	N/A
FTC [25]	70	M	Minimally invasive. No angioinvasion or extrathyroidal extension	8	N/A	N/A	N/A	N/A
FTC [37]	N/A	N/A	Minimally invasive	N/A	N/A	N/A	N/A	N/A
FTC [26]	N/A	N/A	Minimally Invasive	N/A	N/A	N/A	N/A	No
FTC [26]	N/A	N/A	Minimally Invasive	N/A	N/A	N/A	N/A	No
FTC [26]	N/A	N/A	Encapsulated angioinvasive	N/A	N/A	N/A	N/A	No
FTC [12]	N/A	N/A	Widely invasive (extensive vascular invasion)	N/A	N/A	N/A	N/A	TERT C250T
PDTC [14]	N/A	N/A	Multifocal PDTC associated with a focus of the tall cell subtype of PTC harboring the BRAFV600E mutation.	N/A	N/A	N/A	N/A	N/A

N/A: data not available.

## Data Availability

Data sharing not applicable.

## References

[1] SEER Stat Fact Sheets: Thyroid Cancer. Surveillance, Epidemiology, and End Results Program. http://seer.cancer.gov/statfacts/html/thyro.html.

[2] Matrone A., Campopiano M.C., Nervo A., Sapuppo G., Tavarelli M., De Leo S. (2019). Differentiated Thyroid Cancer, From Active Surveillance to Advanced Therapy: Toward a Personalized Medicine. Front. Endocrinol..

[3] Crescenzi A., Baloch Z. (2023). Immunohistochemistry in the pathologic diagnosis and management of thyroid neoplasms. Front. Endocrinol..

[4] Stanciu M., Ristea R.P., Popescu M., Vasile C.M., Popa F.L. (2022). Thyroid Carcinoma Showing Thymus-like Differentiation (CASTLE): A Case Report. Life.

[5] Matrone A., De Napoli L., Torregrossa L., Aghababyan A., Papini P., Ambrosini C.E., Cervelli R., Ugolini C., Basolo F., Molinaro E. (2022). Core Needle Biopsy Can Early and Precisely Identify Large Thyroid Masses. Front. Oncol..

[6] Agrawal N., Akbani R., Aksoy B.A., Ally A., Arachchi H., Asa S.L., Protopopov A. (2014). The Cancer Genome Atlas Research Network. Integrated genomic characterization of papillary thyroid carcinoma. Cell.

[7] Pearson G., Robinson F., Beers Gibson T., Xu B.E., Karandikar M., Berman K., Cobb M.H. (2001). Mitogen-activated protein (MAP) kinase pathways: Regulation and physiological functions. Endocr. Rev..

[8] Liu W., Zhu J., Wu Z., Yin Y., Wu Q., Wu Y., Zheng J., Wang C., Chen H., Qazi T.J. (2023). Insight of novel biomarkers for papillary thyroid carcinoma through multiomics. Front. Oncol..

[9] Torregrossa L., Viola D., Sensi E., Giordano M., Piaggi P., Romei C., Materazzi G., Miccoli P., Elisei R., Basolo F. (2016). Papillary Thyroid Carcinoma With Rare Exon 15 BRAF Mutation Has Indolent Behavior: A Single-Institution Experience. J. Clin. Endocrinol. Metab..

[10] Monti E., Bovero M., Mortara L., Pera G., Zupo S., Gugiatti E., Dono M., Massa B., Ansaldo G.L., Massimo G. (2015). BRAF Mutations in an Italian Regional Population: Implications for the Therapy of Thyroid Cancer. Int. J. Endocrinol..

[11] Parpounas C., Constantinides V. (2023). Advances in Molecular Profiling and Their Potential Influence on the Extent of Surgery in Well-Differentiated Thyroid Carcinoma (WDTC). Life.

[12] Censi S., Cavedon E., Bertazza L., Galuppini F., Watutantrige-Fernando S., De Lazzari P., Nacamulli D., Pennelli G., Fassina A., Iacobone M. (2017). Frequency and Significance of Ras, Tert Promoter, and Braf Mutations in Cytologically Indeterminate Thyroid Nodules: A Monocentric Case Series at a Tertiary-Level Endocrinology Unit. Front. Endocrinol..

[13] Macerola E., Torregrossa L., Ugolini C., Bakkar S., Vitti P., Fadda G., Basolo F. (2017). BRAF(K601E) Mutation in a Follicular Thyroid Adenoma: A Case Report. Int. J. Surg. Pathol..

[14] Giannini R., Ugolini C., Lupi C., Proietti A., Elisei R., Salvatore G., Berti P., Materazzi G., Miccoli P., Santoro M. (2007). The heterogeneous distribution of BRAF mutation supports the independent clonal origin of distinct tumor foci in multifocal papillary thyroid carcinoma. J. Clin. Endocrinol. Metab..

[15] Hwang T.S., Kim W.Y., Han H.S., Lim S.D., Kim W.S., Yoo Y.B., Park K.S., Oh S.Y., Kim S.K., Yang J.H. (2015). Preoperative RAS mutational analysis is of great value in predicting follicular variant of papillary thyroid carcinoma. Biomed. Res. Int..

[16] Kim W.Y., Ko Y.S., Hwang T.S., Han H.S., Lim S.D., Kim W.S., Oh S.Y. (2013). A Case of Multifocal Papillary Thyroid Carcinoma Consisting of One Encapsulated Follicular Variant with BRAF K601E Mutation and Three Conventional Types with BRAF V600E Mutation. Korean J. Pathol..

[17] Leboulleux S., Dupuy C., Lacroix L., Attard M., Grimaldi S., Corre R., Ricard M., Nasr S., Berdelou A., Hadoux J. (2019). Redifferentiation of a BRAF(K601E)-Mutated Poorly Differentiated Thyroid Cancer Patient with Dabrafenib and Trametinib Treatment. Thyroid.

[18] Baloch Z.W., Asa S.L., Barletta J.A., Ghossein R.A., Juhlin C.C., Jung C.K., LiVolsi V.A., Papotti M.G., Sobrinho-Simoes M., Tallini G. (2022). Overview of the 2022 WHO Classification of Thyroid Neoplasms. Endocr. Pathol..

[19] Ciampi R., Romei C., Ramone T., Prete A., Tacito A., Cappagli V., Bottici V., Viola D., Torregrossa L., Ugolini C. (2019). Genetic Landscape of Somatic Mutations in a Large Cohort of Sporadic Medullary Thyroid Carcinomas Studied by Next-Generation Targeted Sequencing. iScience.

[20] Romei C., Tacito A., Molinaro E., Piaggi P., Cappagli V., Pieruzzi L., Matrone A., Viola D., Agate L., Torregrossa L. (2018). Clinical, pathological and genetic features of anaplastic and poorly differentiated thyroid cancer: A single institute experience. Oncol. Lett..

[21] Ganly I., Makarov V., Deraje S., Dong Y., Reznik E., Seshan V., Nanjangud G., Eng S., Bose P., Kuo F. (2018). Integrated Genomic Analysis of Hurthle Cell Cancer Reveals Oncogenic Drivers, Recurrent Mitochondrial Mutations, and Unique Chromosomal Landscapes. Cancer Cell.

[22] Pozdeyev N., Gay L.M., Sokol E.S., Hartmaier R., Deaver K.E., Davis S., French J.D., Borre P.V., LaBarbera D.V., Tan A.C. (2018). Genetic Analysis of 779 Advanced Differentiated and Anaplastic Thyroid Cancers. Clin. Cancer Res..

[23] Pennelli G., Vianello F., Barollo S., Pezzani R., Merante Boschin I., Pelizzo M.R., Mantero F., Rugge M., Mian C. (2011). BRAF(K601E) mutation in a patient with a follicular thyroid carcinoma. Thyroid. Off. J. Am. Thyroid. Assoc..

[24] Afkhami M., Karunamurthy A., Chiosea S., Nikiforova M.N., Seethala R., Nikiforov Y.E., Coyne C. (2016). Histopathologic and Clinical Characterization of Thyroid Tumors Carrying the BRAF(K601E) Mutation. Thyroid. Off. J. Am. Thyroid. Assoc..

[25] Cho U., Oh W.J., Bae J.S., Lee S., Lee Y.S., Park G.S., Lee Y.S., Jung C.K. (2014). Clinicopathological features of rare BRAF mutations in Korean thyroid cancer patients. J. Korean Med. Sci..

[26] Jung C.K., Kim Y., Jeon S., Jo K., Lee S., Bae J.S. (2018). Clinical utility of EZH1 mutations in the diagnosis of follicular-patterned thyroid tumors. Hum. Pathol..

[27] McFadden D.G., Sadow P.M. (2021). Genetics, Diagnosis, and Management of Hurthle Cell Thyroid Neoplasms. Front. Endocrinol..

[28] Christofer Juhlin C., Mete O., Baloch Z.W. (2023). The 2022 WHO classification of thyroid tumors: Novel concepts in nomenclature and grading. Endocr. Relat. Cancer.

[29] Stanciu M., Bera L.G., Popescu M., Grosu F., Popa F.L. (2017). Hashimoto’s thyroiditis associated with thyroid adenoma with Hurthle cells—Case report. Rom. J. Morphol. Embryol..

[30] Gopal R.K., Kubler K., Calvo S.E., Polak P., Livitz D., Rosebrock D., Sadow P.M., Campbell B., Donovan S.E., Amin S. (2018). Widespread Chromosomal Losses and Mitochondrial DNA Alterations as Genetic Drivers in Hurthle Cell Carcinoma. Cancer Cell.

[31] Sinno S., Choucair M., Nasrallah M., Wadi L., Jabbour M.N., Nassif S. (2016). Activating BRAF Mutations Detected in Mixed Hurthle Cell Carcinoma and Multifocal Papillary Carcinoma of the Thyroid Gland: Report of an Unusual Case and Review of the Literature. Int. J. Surg. Pathol..

[32] Cabanillas M.E., Ryder M., Jimenez C. (2019). Targeted Therapy for Advanced Thyroid Cancer: Kinase Inhibitors and Beyond. Endocr. Rev..

[33] Yuan X., Yuan H., Zhang N., Liu T., Xu D. (2022). Thyroid carcinoma-featured telomerase activation and telomere maintenance: Biology and translational/clinical significance. Clin. Transl. Med..

[34] Xing M., Liu R., Liu X., Murugan A.K., Zhu G., Zeiger M.A., Pai S., Bishop J. (2014). BRAF V600E and TERT promoter mutations cooperatively identify the most aggressive papillary thyroid cancer with highest recurrence. J. Clin. Oncol..

[35] Park J.Y., Kim W.Y., Hwang T.S., Lee S.S., Kim H., Han H.S., Lim S.D., Kim W.S., Yoo Y.B., Park K.S. (2013). BRAF and RAS mutations in follicular variants of papillary thyroid carcinoma. Endocr. Pathol..

[36] Jaber T., Waguespack S.G., Cabanillas M.E., Elbanan M., Vu T., Dadu R., Sherman S.I., Amit M., Santos E.B., Zafereo M. (2018). Targeted Therapy iAdvanced Thyroid Cancer to Resensitize Tumors to Radioactive Iodine. J. Clin. Endocrinol. Metab..

[37] Schulten H.J., Salama S., Al-Mansouri Z., Alotibi R., Al-Ghamdi K., Al-Hamour O.A., Sayadi H., Al-Aradati H., Al-Johari A., Huwait E. (2012). BRAF mutations in thyroid tumors from an ethnically diverse group. Hered Cancer Clin. Pract..

